# Pre-Weaning Inulin Supplementation Alters the Ileal Transcriptome in Pigs Regarding Lipid Metabolism

**DOI:** 10.3390/vetsci8100207

**Published:** 2021-09-24

**Authors:** Martine Schroyen, Bing Li, Ester Arévalo Sureda, Yuping Zhang, Julie Leblois, Dieter Deforce, Filip Van Nieuwerburgh, José Wavreille, Nadia Everaert

**Affiliations:** 1 Precision Livestock and Nutrition Laboratory, TERRA Teaching and Research Centre, Gembloux Agro-Bio Tech, University of Liège, B-5030 Gembloux, Belgium; Martine.Schroyen@uliege.be (M.S.); libing881011@163.com (B.L.); Ester.ArevaloSureda@uliege.be (E.A.S.); zhangyuping@caas.cn (Y.Z.); 2 Association Wallonne de l’Élevage asbl (AWÉ), B-5590 Ciney, Belgium; jleblois@awenet.be; 3 Laboratory of Pharmaceutical Biotechnology, Faculty of Pharmaceutical Sciences, Ghent University, B-9000 Ghent, Belgium; Dieter.Deforce@UGent.be (D.D.); Filip.VanNieuwerburgh@UGent.be (F.V.N.); 4 Walloon Agricultural Research Center, Department of Production and Sectors, B-5030 Gembloux, Belgium; j.wavreille@cra.wallonie.be

**Keywords:** inulin, pre-weaning, piglets, ileum, lipid metabolism

## Abstract

Prebiotics, such as inulin, are non-digestible compounds that stimulate the growth of beneficial microbiota, which results in improved gut and overall health. In this study, we were interested to see if, and how, the ileal transcriptome altered after inulin administration in the pre-weaning period in pigs. Seventy-two Piétrain–Landrace newborn piglets were divided into three groups: (a) a control (CON) group (*n* = 24), (b) an inulin (IN)-0.5 group (*n* = 24), and (c) an IN-0.75 group (*n* = 24). Inulin was provided as a solution and administered twice a day. At week 4, eight piglets per group, those closest to the average in body weight, were sacrificed, and ileal scrapings were collected and analyzed using 3′ mRNA massively parallel sequencing. Only minor differences were found, and three genes were differentially expressed between the CON and IN-0.5 group, at an FDR of 10%. All three genes were downregulated in the IN-0.5 group. When comparing the CON group with the IN-0.75 group, five genes were downregulated in the IN-0.75 group, including the three genes seen earlier as differentially expressed between CON and IN-0.5. No genes were found to be differential expressed between IN-0.5 and IN-0.75. Validation of a selection of these genes was done using qRT-PCR. Among the downregulated genes were Angiopoietin-like protein 4 (ANGPTL4), Aquaporin 7 (AQP7), and Apolipoprotein A1 (APOA1). Thus, although only a few genes were found to be differentially expressed, several of them were involved in lipid metabolism, belonging to the peroxisome proliferator-activated receptor (PPAR) signaling pathway and known to promote lipolysis. We, therefore, conclude that these lipid metabolism genes expressed in the ileum may play an important role when supplementing piglets with inulin early in life, before weaning.

## 1. Introduction

Prebiotics are non-digestible compounds that stimulate the growth of beneficial microbiota, which results in the improved gut and overall health of the host [1]. Favorable health-associated microbiota, such as lactobacillus species, preferentially ferment the prebiotic fibers to make a wide range of metabolites, including short chain fatty acids (SCFA). More and more research is focusing on the use of prebiotics, such as inulin, around the critical time of weaning, when piglets are the most susceptible to post-weaning diarrhea (PWD). Studies have shown that adding inulin to the diet of piglets after weaning has the potential to modify the microbial community in different segments of the gastro-intestinal tract [2,3,4], to subsequently increase SCFA production [5], and to alter intestinal permeability, thereby increasing glucose absorption [6]. Moreover, the piglet’s immune response is also altered [7,8], which could result in the reduced incidence and severity of PWD and the shedding of enterotoxigenic *Escherichia coli* [7].

However, rather than focusing on the weaning period, a few studies looked at the supplementation of non-digestible carbohydrates to the feed of newborn piglets. As such, a galacto-oligosaccharide (GOS) prebiotic administered during the first weeks after birth also leads to a shift in microbial community and an increase in several microbial metabolites, endocrine peptides, antimicrobial peptides, and anti-inflammatory cytokines [9], as well as histomorphological changes and increased tight junction proteins [10]. However, not many studies can be found in newborn piglets regarding inulin administration.

Li et al. (2018) [11] investigated growth performance and gut morphology, as well as SCFA production and various specific microbiota in the cecum and colon, in the same piglets as described in this manuscript. The different groups received different concentrations of inulin during the pre-weaning period. From week 3 onwards, supplementation with a specific amount of inulin resulted in a significantly higher body weight of piglet than those not receiving inulin. In addition, the villi/crypt ratio of this inulin group was significantly higher in the jejunum and ileum when compared to the control group. Moreover, an increased production of total SCFA in the colon was seen at week 4, as well as a decreased relative abundance of *Escherichia coli* and Enterobacteriaceae, indicating beneficial effects of inulin administration. Therefore, we were interested to examine the transcriptome of the ileum and to investigate if changes due to inulin administration would result at the level of gene expression. We hypothesized that due to the early administration of inulin significant changes in the ileum transcriptome would occur, since it has been noted to improve gut health, increase the presence of beneficial microbiota, and increase growth.

## 2. Materials and Methods

### 2.1. Animals and Sampling

The animal experiment was approved by the ethical committee of the University of Liège (protocol n°1640). The experimental setup was previously described by Li et al. (2018) [11]. In short, 72 Piétrain–Landrace suckling piglets, 6 piglets from 12 litters with an average of 12 piglets per litter were used. Piglets were selected based on their initial body weight and equally divided into three groups, taking into account sex, body weight, and the sow’s parity: (a) a CON group, which did not receive inulin, but received a sham solution; (b) an IN-0.5 group, which received inulin supplementation at 0.50 g per day in week 1, increasing by 0.50 g per week till week 4; and (c) an IN-0.75 group, which received inulin supplementation at 0.75 g per day in week 1, and increasing by 0.75 per week till week 4. Inulin was provided by Cosucra (Warcoing, Belgium). Supplementation was done using deionized aqueous solutions, with 0%, 20%, or 30% inulin for the CON group, the IN-0.5 group, and the IN-0.75 group, respectively, which were orally administered to the piglets using a syringe via the mouth, twice a day, at 09:00 and 15:00. Since inulin is known to change the gut microbiome, the entire litter received the same treatment, to avoid cross-contamination. Per treatment, four litters received supplementation. Animals were housed at the Walloon Agricultural Research Center (Gembloux, Belgium). Ambient temperature was maintained at 28 °C. Twenty-four piglets, those that were the closest to the average body weight per group and with respect to having an equal division in sex, were anesthetized by isoflurane before euthanasia by exsanguination at 28 days of age. Ileal scrapings were collected by scraping the mucosal layer of a midpiece of the ileum with a microscope glass slide. These scrapings were snap frozen in liquid nitrogen and stored at −80 °C, prior to RNA extraction. We were able to extract RNA from ileal scrapings from 7 CON animals, 8 IN-0.5 animals, and 8 IN-0.75 animals.

### 2.2. Transcriptome Analysis

RNA was extracted using a ReliaPrep RNA Tissue Miniprep System kit (Promega, Madison, WI, USA) according to the manufacturer’s protocol. Quantity and quality were checked using an Agilent Bioanalyzer 2100 (Agilent Technologies Inc., Santa Clara, CA, USA). A QuantSeq 3′ mRNA-seq library prep kit (Lexogen, Vienna, Austria) was used to sequence the mRNA from the 3′ end on a Nextseq500 (Illumina, San Diego, CA, USA). The reads were mapped on the Ensembl Sus scrofa reference genome version 11.1 using STAR mapping software [12]. Quality metrics were computed using FastQC [13]. DESeq2 was used to find differential expression [14]. The pathway and gene ontology (GO) term analyses were made using g:profiler [15].

### 2.3. qRT-PCR Validation

Validation of three genes (ANGPTL4, APOA1, and AQP7), tested relative to two housekeeping genes (beta-actin (ACTB) and glyceraldehyde 3-phosphate dehydrogenase (GAPDH)), was done by qPCR performed on a LightCycler^®®^ 480 Instrument II (Roche, Basel, Switzerland) using SYBR Premix Ex Taq Tli RNAse H Plus (TakaraBio, Kusatsu, Japan). RNA was reverse transcribed using GoScript TM Reverse Transcription Mix (Promega, Madison, WI, USA), following the manufacturer’s instructions. Primers were found in the literature or designed using Primer3 [16] (Table 1).

The program used was a standard program with denaturation at 95 °C for 5 s, followed by 40 cycles of annealing at 60 °C for 30 s and elongation at 72 °C for 45 s. Primer efficiencies were between 100% and 110%, and specificity was checked through melting curve analyses. A general linear model was performed with treatment as the variable, comparing the CON group with the two inulin groups using SAS 9.4 (SAS Inc., Cary, NC, USA). Significant group means were determined by Tukey’s range test.

### 2.4. Statistical Analyses

The statistical analysis performed to find differential expression by RNA-seq was done using DESeq2 [14]. Correction for multiple testing was done using the Benjamini–Hochberg correction method, which is the default *p*-value adjustment method embedded in DESeq2. For our analysis, an adjusted *p*-value or false discovery rate (FDR) threshold of 10% was used. For the statistical analysis of the qRT-PCR results, a general linear model was performed with treatment as the variable, comparing the CON group with the two inulin groups using SAS 9.4 (SAS Inc.). Significant group means were determined by Tukey’s range test.

## 3. Results

### 3.1. Differentially Expressed Genes

A total of 18,170 transcripts could be mapped to the *Sus scrofa* reference of Ensembl. Of these genes, only three were differentially expressed between the CON group and the IN-0.5 group. All of them were downregulated in the IN-0.5 group (Table 2). When comparing the CON group with IN-0.75 group, eight genes were differentially expressed. Five of them were downregulated in the IN-0.75 group, including the three genes seen earlier as differentially expressed between CON and IN-0.5. Three genes were upregulated in the IN-0.75 group compared to the CON group (Table 2). No genes were found to be differential expressed between IN-0.5 and IN-0.75. We validated three genes, Angiopoietin-like protein 4 (ANGTPL4), Apoliprotein A1 (APOA1), and Aquaporin 7 (AQP7), using qPCR. Their expression was measured relatively to the geometrical mean of the expression of two housekeeping genes, ACTB and GAPDH.

In Table 3, you can find the relative expression in all experimental groups and the *p*-value comparing the different groups in a one-way ANOVA analysis. For AQP7 the CON group was significantly different from IN-0.5 (*p* = 0.02) and IN-0.75 (*p* = 0.02), while for ANGPTL4 (pCON-IN0.5 = 0.10, pCON-IN0.75 = 0.05) and APOA (pCON-IN0.5 = 0.10, pCON-IN0.75 = 0.12) a trend towards significance could be noted. Therefore, we can state that the qRT-PCR results confirmed the RNA-seq data.

### 3.2. Gene Ontology (GO) Analysis of Differential Expressed Genes

**Figure 1:**
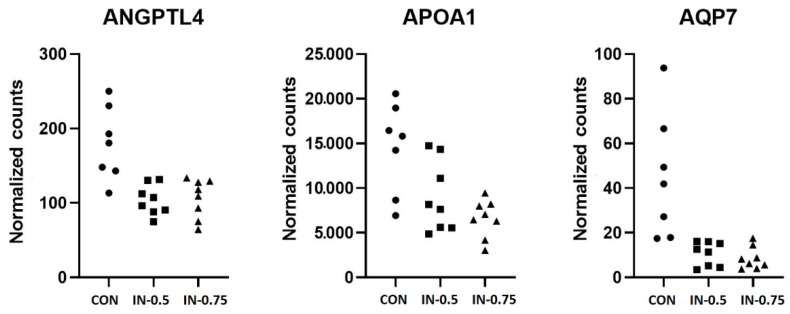
Normalized expression values from RNA-seq for the genes downregulated in the IN-0.75 group compared to the CON group and belonging to the PPAR pathway.

Two of the three genes whose expression was downregulated due to IN-0.5 belonged to the peroxisome proliferator-activated receptor (PPAR) signaling KEGG pathway, namely ANGPTL4 and AQP7. When looking at the differentially expressed genes between the IN-0.75 group and the CON group, APOA1 was added to the differentially expressed genes also belonging to the PPAR signaling pathway. Whereas for ANGPTL4 and AQP7 there was an equally low expression for both inulin treatments compared to the control, for APOA1 a dose dependent expression pattern could be observed, as reflected by the differential expression analysis of the RNA-seq (Figure 1).

## 4. Discussion

Although only a few genes were found to be differentially expressed in the ileum due to the early administration of inulin in pigs, a significant part of these genes are present in a specific pathway, namely the PPAR signaling pathway. Indeed, it has been previously reported that prebiotics such as inulin can have an effect on lipid metabolism [21,22]. In mice, inulin supplementation after receiving a high-fat diet resulted in a downregulation of the PPAR signaling pathway in adipose tissue, compared to not supplementing with inulin [23]. In our study, ANGPTL4, APOA1, and AQP7, all involved in lipid metabolism and part of the PPAR signaling pathway, were downregulated in the ileum of the inulin treated groups. Inulin administration is accompanied by an elevation in SCFA production, as these metabolites are derived from the intestinal fermentation of carbohydrates by microbiota [24]. We also observed in our study, in the IN-0.5 group, an increase in total SCFA concentration and in iso-butyrate in the colon, as well as propionate and iso-butyrate in the cecum [11]. SCFAs have been noted to activate the G protein-coupled receptors GPR41 and GPR43, also known as free fatty acid receptors, which are expressed in those cells exposed to the highest concentrations of SCFA, such as the ileum, colon, and adipocytes [25]. Interestingly, the activation of GPR43 by acetate or propionate exhibits a reduction in lipolytic activity in adipocytes and suppresses free fatty acids in the plasma [26]. We hypothesize that the expression of ANGPTL4, APOA1, and AQP7 in the intestine is decreased as a result of the increase in SCFA produced by beneficial bacteria due to the supplementation of inulin. However, although, based on the literature, we assume that inulin stimulates the production of SCFA, it cannot be excluded that the microbiota directly, or other microbial metabolites, induced these changes in gene expression. Furthermore, the question remains of whether these genes contributed directly or indirectly to the higher body weight seen in the inulin supplemented group (IN-0.5).

ANGPTL4 is a lipoprotein lipase inhibitor involved in both lipid metabolism and angiogenesis [27]. It is secreted by entero-endocrine cells and has been proposed as a mediator between gut microbiota and fat storage in adipose tissue [28], by inhibiting the circulating lipoprotein lipase that regulates the uptake of triglycerides in adipocytes [29]. Gut microbiota act through suppression of ANGPTL4 in the small intestinal epithelium [29,30]. By attenuating the intestinal expression of ANGPTL4, microbiota, therefore, promote lipoprotein lipase controlled fatty acid storage in adipose tissue [31], which may, thus, have occurred in our study as well. Interestingly, Alex et al. (2014) [28] noted that probiotic strains were able to stimulate ANGPTL4 expression in several enterocyte cell lines.

APOA1 is a component of high-density lipoprotein (HDL) and therefore often used as marker for cardiovascular diseases [32]. HDL is synthesized and secreted into circulation by the liver, and thus APOA1 is most highly expressed in the liver [33]. The expression levels of APOA1 in the liver have been reported to be affected by SCFA from gut microbiota [34,35]. In the colon of mice, APOA1 was found to be less highly expressed when comparing groups undergoing a fecal microbiota transplant or receiving two different Lactobacillus strains with a control group; which was explained as a consequence of the immune defense function of APOA1 [36]. Indeed, APOA1 has been shown to play a role in inflammation and immune response [37,38]. Dunislawska et al. (2021) reported that chickens receiving the prebiotic inulin in ovo had a decreased APOA1 expression over time, which was hypothesized as being the result of digestive and metabolic adaptations [39]. These decreased levels of APOA1 could also mean that anti-inflammatory functions are weakened in favor of pro-inflammatory reactions [39]. In our study, we saw a decrease of APOA1 expression in the ileum that was dose-dependent and significantly different from the control when given at the higher amount of 0.75 g per day in week 1 and increasing by 0.75 per week up to week 4. However, its role in lipoprotein metabolism or immune response in this study remains unknown.

AQP7 is a glycerol transporting protein, regulating glycerol efflux in adipocytes, and thereby influencing lipid and glucose metabolism [40]. In the intestine, AQP7 has been suggested to play a role in fluid absorption and secretion [41]. However, Vieira da Silva et al. (2021) investigated the influence of glutamine or cysteine as feed supplementations and observed a significant downregulation of AQP7 in the ileum, without an impact on water permeability [42]. Unlike classical aquaporins that are selective to water, AQP7 is an aquaglyceroporin, which allows transport of small molecules such as glycerol; the regulation of gut membrane glycerol permeability is crucial to control fat deposition, lipolysis, and gluconeogenesis [42]. In the human small intestine, reduced levels of AQP7 mRNA were correlated with inflammatory bowel diseases such as Crohn’s disease or ulcerative colitis [43]. In our study, we observed a downregulation of AQP7 in the inulin treated groups in the ileum, which may have affected glycerol permeability.

Other genes that were downregulated due to at least one dose of the inulin supplementation were ATPase H+ Transportin V1 subunit C2 (ATP6V1C2) and Glycerophosphocholine Phosphodiesterase 1 (GPCPD1). To date, they have not been described in the context of feed supplementation, prebiotic administration, or microbial fermentation. Genes upregulated due to the inulin supplementation where only upregulated when the highest dose of IN-0.75 was given, i.e., Protein C receptor (PROCR), clavesin 2 (CLVS2), and nucleoside-triphosphate (NTPCR). However, so far, none of these genes have been described in the context of feed supplementation.

In conclusion, even though not many transcriptomic changes were found in the ileum of piglets given inulin in the first few weeks of their life, a few genes important in lipid metabolism were significantly downregulated. More knowledge of the ileal microbiota and metabolite composition could help unravel their role in this transcriptomic change.

## Figures and Tables

**Table 1:** Primers used for qRT-PCR validation.

Gene	Primer Sequence (5′ → 3′)	Reference
ANGPTL4	F: GGAGAAGCAGCACTTGAGAA	Ren et al., 2014 [17]
	R: GGGTCATCTTGGGTAGTCTTT	
APOA1	F: GCAAGATGACCCGCAGTCACC	Uribe et al., 2016 [18]
	R: GCCACTGTCTTTGATCGCATCC	
AQP7	F: GTTTGGTCTAGGCTCCGTGG	Own design
	R: GGTCACTGTCAGCTTTCCCT	
ACTB	F: TCTGGCACCACACCTTCT	Su et al., 2018 [19]
	R: TGATCTGGGTCATCTTCTCAC	
GAPDH	F: AATGGGGTGATGCTGGTGCT	Lee and Kang, 2017 [20]
	R: GGCAGAAGGGGCAGAGATGA	

**Table 2:** Differentially expressed genes in the comparisons between the CON group and the IN-0.5 group or the CON group and the IN-0.75 group at a Benjamini–Hochberg false discovery rate (FDR) of 0.10. log2FC is log2 fold change, SE log2FC is the standard error of the log2FC. *n* = 7 for CON, *n* = 8 for IN-0.5, and *n* = 8 for IN-0.75.

**CON Versus IN-0.50 at an FDR of 0.10**						
**Ensembl Gene ID**	**Gene Symbol**	**Average Expression**	**log2FC**	**SE log2FC**	***p*** **-Value**	**FDR**
ENSSSCG00000013599	ANGPTL4	127.38	0.8	0.18	7.56 × 10^−6^	0.05
ENSSSCG00000010992	AQP7	19.83	2.16	0.41	1.96 × 10^−7^	0.004
ENSSSCG00000008634	ATP6V1C2	9.8	2.87	0.64	8.26 × 10^−6^	0.05
**CON Versus IN-0.75 at an FDR of 0.10**						
**Ensembl Gene ID**	**Gene Symbol**	**Average Expression**	**log2FC**	**SE log2FC**	***p*** **-Value**	**FDR**
ENSSSCG00000026585	PROCR	9.11	−1.91	0.45	1.97 × 10^−5^	0.071
ENSSSCG00000004232	CLVS2	23.37	−1.88	0.34	4.59 × 10^−8^	0
ENSSSCG00000010166	NTPCR	16.45	−1.04	0.25	3.70 × 10^−5^	0.084
ENSSSCG00000007043	GPCPD1	170.23	0.47	0.11	2.83 × 10^−5^	0.073
ENSSSCG00000013599	ANGPTL4	127.38	0.75	0.18	2.34 × 10^−5^	0.071
ENSSSCG00000030921	APOA1	9840.34	1.14	0.26	1.45 × 10^−5^	0.066
ENSSSCG00000010992	AQP7	19.83	2.47	0.42	4.52 × 10^−9^	0
ENSSSCG00000008634	ATP6V1C2	9.8	3.19	0.66	1.23 × 10^−6^	0.007

Angiopoietin Like 4 (ANGPTL4); Aquaporin 7 (AQP7); ATPase H+ Transportin V1 subunit C2 (ATP6V1C2); protein C receptor (PROCR); clavesin 2 (CLVS2), nucleoside-triphosphate (NTPCR); Glycerophosphocholine Phosphodiesterase 1 (GPCPD1).

**Table 3:** Validation of differentially expressed genes in the comparisons between the CON group and the IN-0.5 group or the CON group and the IN-0.75 group by qPCR. Numbers are relative expression values in relation to the geometrical mean of the expression of ACTB and GAPDH. One-way ANOVA was used to obtain the *p*-value. *n* = 7 for CON, *n* = 8 for IN-0.5, and *n* = 8 for IN-0.75.

Ensembl Gene ID	Gene Symbol	Expression CON	Expression IN-0.5	Expression IN-0.75	*p*-Value
ENSSSCG00000013599	ANGPTL4	1.86 ± 0.36	1.02 ± 0.20	1.03 ± 0.25	0.071
ENSSSCG00000030921	APOA1	1.26 ± 0.20	0.76 ± 0.12	0.66 ± 0.17	0.044
ENSSSCG00000010992	AQP7	1.32 ± 0.20	0.41 ± 0.12	0.38 ± 0.17	0.011

Angiopoietin Like 4 (ANGPTL4); Apolipoprotein A1 (APOA1); Aquaporin 7 (AQP7).

## Data Availability

All raw RNAseq sequences were submitted to the European Nucleotide Archive database under the accession number PRJEB44896 and will be made accessible after publication.
